# 

**Published:** 1868-10

**Authors:** 


### Books and Pamphlets Received.

A Handbook of Vaccination. By Edward C. Seaton, M. D., Medical Inspector to the Privy Council. Philadelphia: J. B. Lippincott & Co., 1868.

Conservative Surgery in its General and Successful Adaptation in cases of Severe Traumatic Injuries of the Limbs, with a Report of Cases. By Albert G. Walter, M. D. Pittsburgh: W. G. Johnson & Co.

Transactions of the Twenty-third Annual Meeting of the Ohio State Medical Society, held at Delaware, June 2, 3, 4, 1868.

Transactions of the Eighteenth Anniversary Meeting of the Illinois State Medical Society, held in Quincy, May 19 and 20, 1868.

Atlas of Venereal Diseases. Part IV. By Freeman J. Bumstead, M. D. Philadelphia: Henry C. Lea.

A Sermon delivered March 26, 1868, at the funeral of the Hon. Henry Halsey Childs, M. D., at Pittsfield, Mass., by John Todd, D. D.

MARRIED—At Jamestown, on the 29th ult., at the residence of Mrs. M. Burnell, Third street, by Rev. L. W. Norton, CHARLES S. HAZELTINE, M. D., and Miss ELLA A., second daughter of the late Madison Burnell, Esq., all of Jamestown.

